# Diagnosis of dementia in residential aged care settings in Australia: An opportunity for improvements in quality of care?

**DOI:** 10.1111/ajag.12580

**Published:** 2018-09-06

**Authors:** Suzanne M Dyer, Emmanuel S Gnanamanickam, Enwu Liu, Craig Whitehead, Maria Crotty

**Affiliations:** ^1^ Department of Rehabilitation Aged and Extended Care Flinders Medical Centre Flinders University Adelaide South Australia Australia; ^2^ Cognitive Decline Partnership Centre The University of Sydney Sydney New South Wales Australia; ^3^ Mary MacKillop Institute for Health Research Australian Catholic University Melbourne Victoria Australia

**Keywords:** cognitive dysfunction, dementia, diagnosis, quality of health care, residential facilities

## Abstract

**Objective:**

To examine the cognitive status of Australians living in residential aged care facilities (RACFs) and whether or not a dementia diagnosis was recorded.

**Methods:**

Cross‐sectional study of 541 residents of 17 RACFs spanning four states. Examination of cognitive status by Psychogeriatric Assessment Scale Cognitive Impairment Scale (PAS‐Cog) and dementia diagnosis from medical records.

**Results:**

The study population included 65% of residents with a diagnosis of dementia recorded, and 83% had a PAS‐Cog score of four or more indicating likely cognitive impairment. More than 20% of participants had likely cognitive impairment (PAS‐Cog ≥4), but no diagnosis of dementia; 11% had moderate‐to‐severe cognitive impairment (PAS‐Cog ≥10) but no recorded dementia diagnosis.

**Conclusion:**

There may be a lack of formal diagnosis of dementia in Australian RACFs. Greater efforts from all health professionals to improve diagnosis in this setting are required. This is an opportunity for improved person‐centred care and quality of care in this vulnerable population.

**Practice Impact**

There may be a lack of formal diagnosis of dementia in Australian residential aged care facilities. A more coordinated effort from all health professionals to improve diagnosis in this setting could lead to improved person‐centred care and quality of care for this vulnerable population.

## Introduction

Questions around the quality of care in Australian residential aged care facilities (RACFs), particularly for residents with dementia, are topical 1, 2. Providing high‐quality services for these residents is complex, and the media tend to focus on regulations. However, a key component of providing high‐quality care is a person‐centred approach, which requires a detailed understanding of the care needs of every resident.

Australian clinical practice guidelines and principles of care for people with dementia emphasise the importance of a timely diagnosis and recommend that ‘symptoms should be explored when first raised, noted or reported’ 3. However, more than half of people living with dementia in RACFs may lack a formal diagnosis 4. The diagnosis of dementia is often delayed, and its value may be questioned, particularly in a residential care setting 5. However, following the principles of person‐centred care requires knowledge of the full health and personal characteristics of all residents.

In a large cross‐sectional study of Australian residents of aged care facilities, we have conducted an analysis of the cognitive status of the residents and whether or not a dementia diagnosis was recorded.

## Methods

This study was approved by the Flinders University Social and Behavioural Ethics Committee (6732, 6753). The INSPIRED (Investigating Services Provided in the Residential Environment for Dementia) study is a cross‐sectional study including 541 residents from 17 RACFs in Australia spanning four Australian states (New South Wales, Western Australia, Queensland and South Australia) and both rural and metropolitan settings. Details of the study design have been published elsewhere 6, 7, 8. The study was conducted to examine alternative models of care, quality of life and costs of people living with cognitive impairment and dementia in residential care. Facilities were purposely sampled to include those with a high number of residents with dementia and providing novel models of care. This study included 14 not‐for‐profit facilities with a dementia‐specific unit, wing, or facility. Data on participant comorbidities and characteristics were obtained, including Psychogeriatric Assessment Scales Cognitive Impairment Scale (PAS‐Cog) assessments conducted within three months of participant enrolment (or latest available assessment if PAS‐Cog score ≥ 18). Where the PAS‐Cog had been performed more than three months prior to enrolment and was less than 18 on last assessment, a PAS‐Cog assessment was conducted by trained study personnel. A PAS‐Cog score cannot be used to diagnose dementia; however, it is routinely used in Australia as a component of government Aged Care Assessment Team (ACAT) assessments of the care needs of older adults and therefore is the most readily available estimate of the cognitive status of the residents.

Data on dementia diagnosis were collected from the participants' medical records.

## Results

Descriptive characteristics of the study population are presented in Table 1. The study population was broadly similar to that for the general population in residential care Australia‐wide 8. However, there was a greater proportion of residents living in metropolitan areas, living in medium to large facilities, and women. Sixty‐five percent of participants had a diagnosis of dementia recorded, the mean PAS‐Cog score was 13.3 (Standard deviation 7.7) and 83% had a PAS‐Cog score of 4 or more indicating likely cognitive impairment.

**Table 1:** Cognition and baseline characteristics of INSPIRED study sample

Characteristics	*n* (%)/Mean (SD)
Age, mean (SD)	85.5 (8.5)
Female, *n* (%)	403 (75)
Married, *n* (%)	137 (25)
Modified Barthel Index, mean (SD)	40.4 (32.8)
Number of comorbid disease groups (Cohen‐Mansfield Index), mean (SD)	3.7 (1.4)
Recorded medical diagnosis of dementia	348 (64.6)
PAS‐Cog, mean (SD)	13.3 (7.7)
PAS‐Cog 0–< 4	93 (17.2)
PAS‐Cog 4–<10	100 (18.5)
PAS‐Cog 10–<16	82 (15.1)
PAS‐Cog 16–21	266 (49.2)
PAS‐Cog total ≥4	448 (82.8)
Neuropsychiatric Inventory Questionnaire score, mean (SD)	8.3 (6.4)
Tertiary education, *n* (%)	37 (7)
Weekly social interactions with relatives and friends, *n* (%)	378 (70)

PAS‐Cog, Psychogeriatric Assessment Scales Cognitive Impairment Scale; SD, standard deviation.

When we examined the relationship between PAS‐Cog scores and whether or not dementia diagnosis was recorded in the medical records, we found a high prevalence of likely cognitive impairment without an associated dementia diagnosis recorded. More than 20% of participants had likely cognitive impairment as indicated by the PAS‐Cog score, but no diagnosis of dementia (Table 2). Eleven percent had moderate‐to‐severe cognitive impairment as indicated by a PAS‐Cog score of 10 or greater but no recorded dementia diagnosis (Table 2).

**Table 2:** Cognitive and dementia status of INSPIRED study sample

Characteristics	*n* (%)
No diagnosis of dementia recorded and PAS‐Cog 4–<10	61 (11)
No diagnosis of dementia recorded and PAS‐Cog 10–<16	30 (6)
No diagnosis of dementia recorded and PAS‐Cog 16–21	28 (5)
No diagnosis of dementia recorded and PAS‐Cog total ≥4	119 (22)
Diagnosis of dementia recorded and PAS‐Cog 0–<5	21 (4)

PAS‐Cog, Psychogeriatric Assessment Scales Cognitive Impairment Scale.

## Discussion

This study has indicated that up to one‐fifth of residents in Australian RACF settings may have dementia which has not been formally diagnosed. Facilities with a high prevalence of residents with cognitive impairment were specifically recruited, and hence, this proportion is likely to be an overestimate of the rate in the broader Australian RACFs. However, the facilities were also owned by providers that were considered high quality 9. A similar proportion of recent admissions to Norwegian nursing homes were reported to be lacking a diagnosis in the medical records 10. In that study, 83 percent of those recently admitted that met ICD‐10 criteria for a dementia diagnosis, but did not have a diagnosis in the facility records, had moderate or severe dementia.

A systematic review has estimated the rate of undiagnosed dementia in both community and residential care settings worldwide as 62% 4. However, the rates varied widely between studies, due to the different settings, countries, year and methods of dementia diagnosis. None of the included studies were conducted in a residential care setting in Australia. While the PAS‐Cog score cannot provide an estimate of the rate of undetected dementia in our study, the findings highlight a likely need for improved diagnosis of dementia in Australian RACF settings.

Dementia is considered a national health priority area in Australia, with the need for timely diagnosis being a priority area for action 11. The first recommendation in the Australian guidelines and principles of care for people living with dementia is that health and aged care professionals should provide person‐centred care 12. Previous studies have reported improvements in quality of life and agitation for people with dementia in residential care with a person‐centred care and a person‐centred environment approach 13. Another guideline recommendation states that the impact of behavioural and psychological symptoms of dementia can be reduced with a person‐centred care approach 3, 12. However, if we are going to follow the principles of person‐centred care, how can this be done without the basic process of making a diagnosis?

It is likely that for some of the residents in this study, particularly those with moderate or severe cognitive impairment, there is an awareness by staff and/or families that the resident is likely to have dementia; however, a formal diagnosis is not pursued. It may be perceived that there is little value in a diagnosis, particularly for someone elderly living in a RACF 5. However, there are important benefits of a clear diagnosis for this population. The process of diagnosis itself should lead to a more thorough and comprehensive management plan including investigation of associated comorbidities and psychiatric conditions 3. Alternative causes of cognitive problems, such as adverse drugs reactions and depression, should be ruled out. There may also be a belief that there is a lack of value in a diagnosis as there are is no definitive treatment available. However, formal diagnosis is necessary for access to medications (acetylcholinesterase inhibitors or memantine) which delay functional decline in dementia 14. Better management of cardiovascular risk factors for those with vascular dementia may slow progression 15. Diagnosis is also likely to lead to consideration of more appropriate management options, including those for the management of behavioural and psychological symptoms 3, 12. Residents with dementia are less likely to report symptoms of other conditions and may require support in following treatments such as taking medications. Recording a diagnosis of dementia is also likely to ensure that family members are appropriately involved in necessary care decisions.

While undertaking the formal diagnostic process may be difficult in RACFs, some simple strategies could be explored. For example, ACAT approvals often involve careful cognitive assessment but this information may not be available to general practitioners in RACFs. Provision of the details of the ACAT assessment by RACF managers to consulting general practitioners may assist in the process. Lack of communication between nursing staff and primary care physicians has been reported as contributing to a lack of formal diagnosis 10. Where cognitive impairment has been noticed following admission to care, more systematic follow‐up including cognitive assessment is needed 16.

## Conclusion

If a diagnosis of dementia is delivered within a shared decision‐making framework where residents and families are able to participate in future planning, diagnosis is likely to result in improved person‐centred care and quality of care for Australians living with dementia in RACFs. We believe that without better diagnosis of dementia in the aged care sector, optimal person‐centred care is not possible – a more coordinated effort from all health professionals is needed.
